# Multisensory System for Fruit Harvesting Robots. Experimental Testing in Natural Scenarios and with Different Kinds of Crops

**DOI:** 10.3390/s141223885

**Published:** 2014-12-11

**Authors:** Roemi Fernández, Carlota Salinas, Héctor Montes, Javier Sarria

**Affiliations:** 1 Centre for Automation and Robotics (CAR) CSIC-UPM, Ctra. Campo Real, Km. 0,200, La Poveda. Arganda del Rey, Madrid 28500, Spain; E-Mails: carlota.salinas@car.upm-csic.es (C.S.); hector.montes@car.upm-csic.es (H.M.); javier.sarria@car.upm-csic.es (J.S.); 2 Faculty of Electrical Engineering, Technological University of Panama, Panama City 0819, Panama

**Keywords:** precision agriculture, fruit detection, multisensory system, time-of-flight camera, multispectral system, optical filters

## Abstract

The motivation of this research was to explore the feasibility of detecting and locating fruits from different kinds of crops in natural scenarios. To this end, a unique, modular and easily adaptable multisensory system and a set of associated pre-processing algorithms are proposed. The offered multisensory rig combines a high resolution colour camera and a multispectral system for the detection of fruits, as well as for the discrimination of the different elements of the plants, and a Time-Of-Flight (TOF) camera that provides fast acquisition of distances enabling the localisation of the targets in the coordinate space. A controlled lighting system completes the set-up, increasing its flexibility for being used in different working conditions. The pre-processing algorithms designed for the proposed multisensory system include a pixel-based classification algorithm that labels areas of interest that belong to fruits and a registration algorithm that combines the results of the aforementioned classification algorithm with the data provided by the TOF camera for the 3D reconstruction of the desired regions. Several experimental tests have been carried out in outdoors conditions in order to validate the capabilities of the proposed system.

## 1. Introduction

Service robots are becoming a key part of many sectors of the society, including precision agriculture, where they are called to play an important role in improving competitiveness and sustainable production [1]. Precision agriculture oriented to the automatic harvesting of fruits requires the investigation of non-destructive sensors capable of collecting precise and unambiguous information for an efficient detection and localization of fruits. This task of detection and localisation in natural scenes is quite challenging, since most fruits are partially occluded by leaves, branches or overlapped with other fruits [2]. These occlusions eliminate the direct correspondence between visible areas of fruits and the fruits themselves by introducing ambiguity in the interpretation of the shape of the occluded fruit [3]. In addition, colours of fruits cannot be rigidly defined because the high variability exhibited among the different cultivars within a same species and the different levels of ripeness. Moreover, fruits can be found in quite random positions and orientations in trees of various sizes, volumes and limb structures. Environmental conditions such as wind, rain, dust, moisture and lighting also increase the technical challenge imposed to the sensory system [4].

Given the strong dependence of the fruit harvesting robots on sensorial information, and the numerous problems to be solved in this area due to the application requirements, there has been an intensive research effort during the last four decades, aiming to provide automatic detection and localisation of fruits. Most of the related studies reported in the literature are based on the use of computer vision and other image processing techniques. One of the first studies was presented by [5], who identified from their measurements that the surface of oranges reflected ten times more light than the leaves. In [6] the first computer vision system for detecting apples and guiding a harvesting robot was implemented. The proposed system was based on a monochrome camera and a red optical filter to increase the contrast between red apples and green-coloured leaves. In [7] a vision system based on a single colour camera was proposed for the tomato harvesting Agrobot robotic system. Hue and saturation histograms were employed to perform thresholding to segment the image whereas the 3D information was obtained by stereo-matching of two different images of the same scene. Two approaches based on colour information to solve the fruit recognition problem for a citrus picking robot were presented in [8,9]. A system based on a monochrome camera to detect and located tomatoes in natural settings was also developed in [10]. Each acquired image was processed in order to find circular arcs that could correspond to tomato contours. The automatic detection of apples by using a stereo vision system which provided the 3D-dimensional position of each detected fruit was addressed in [11]. A sensory system based on an infrared laser range-finder sensor that provided range and reflectance images, capable of detecting spherical fruits in non-structured environments was designed and implemented in [12]. Some comprehensive reviews like [4,13] cover several aspects of these and other not-mentioned-systems.

More recently, Van Henten, *et al.* [14] achieved a high detection rate of cucumber fruits by combining the images acquired by two cameras, one equipped with an 850 nm filter and the other with a filter in the 970 nm band. Bulanon, *et al.* [15] used a real time machine vision system based on a colour CCD camera to determine the location of the apples centres and the abscission layer of the peduncles. In a later approach, Bulanon and Kataoka [16] extended their earlier study by combining the machine vision system based on a colour CCD camera with a laser ranging sensor to determine the distance to the fruit. Tanigaki, *et al.* [17] designed and manufactured a 3D vision system that has two laser diodes for a cherry-harvesting robot. One of these laser diodes emits a red beam and the other an infrared beam. The 3D shape of the cherries was measured by scanning the laser beams, and the red fruits were distinguished from other objects by the difference in the spectral-reflection characteristics between the red and infrared laser beams. A multispectral analysis was also carried out in [18] to enhance citrus fruit detection in the field. In [19,20] authors proposed a machine vision unit that consists of three aligned CCD cameras for guiding a strawberry-harvesting robot. In this case, the two side cameras were used to provide stereo vision to determine the fruit position in the 3D space, while a camera located in the centre was used to detect the peduncle and to calculate its inclination.

All the studies mentioned above are limited to fruit detection. Nevertheless, for the harvesting task, it would be advantageous to detect and localise other plant elements that could interference in the free motion of the robotic manipulator. In [21] Cabernet Sauvignon grapevine elements are discriminated for precision viticulture tasks such as harvesting, whereas in [22] the problem of plant parts detection is addressed for the motion planning of a sweet-pepper harvesting robot. Also worthy of mention are the researches carried out by [23,24]. Although the sensory systems proposed in these studies have not been designed for harvesting robots, they addressed the detection and localization of plant elements for other precision agriculture tasks as selective spraying and yield estimation.

This paper presents the research carried out in order to assess the feasibility of detecting, discriminating and locating fruits and other plant elements from different kinds of crops in natural environments by utilising a unique modular and easily adaptable multisensory system and a set of associated pre-processing algorithms. The proposed solution is intended to be used in autonomous harvesting robotic systems, without requiring previous preparation of the crops.

## 2. Materials and Methods

This section describes the automatic multisensory rig that has been designed for the data acquisition and explains the pre-processing algorithms that has been implemented for the proposed multisensory system. A validation strategy is also presented for evaluating qualitatively the system performance.

### 2.1. Multisensory System Description

All harvesting robots require a sensory system that provides reliable data that can be processed and analysed in order to detect the presence of fruits, discriminate them from the rest of the scene elements and locate them spatially. In addition to complying with these fundamental objectives necessary for the efficient performance of the harvesting robot, the sensory system proposed in this study also intends to offer modularity, versatility and adaptability, so that the same rig can be utilised in various settings and with different types of crops without requiring major modifications.

**Figure 1.**
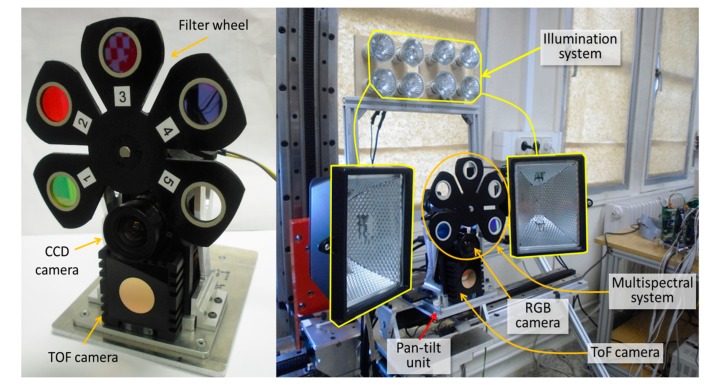
Close-up views of the multisensory system for fruit harvesting robots.

The proposed multisensory system consists of an AVT Prosilica GC2450 high resolution CCD colour camera, a multispectral imaging system and a Mesa SwissRanger SR-400011 TOF 3D camera [25]. The 5-megapixel GC2450 has a frame rate of up to 15 fps at 2448 × 2050 pixels resolution. Meanwhile, the TOF camera provides a depth map and amplitude image at the resolution of 176 × 144 pixels with 16 bit floating-point precision, as well as *x, y* and *z* coordinates to each pixel in the depth map. The detection range (radial distances) of this device goes from 0.1 m to 5.0 m, and its field of view is 69° (h) × 56° (v). The high resolution colour camera is not only utilised for the acquisition of RGB images, but also as part of the multispectral system, in which case it is set in the monochrome mode. The multispectral system is completed with a custom-made filter wheel and a servomotor that is responsible for the accurate positioning of the filter wheel. This positioning can be achieved with a maximum angular velocity of 40 rpm and a position error if 0.001°. The filter wheel allows interchanging up to five optical filters, facilitating the adaptation of the system for the detection of different kinds of crops. Since correct illumination could be critical in some scenarios, the system also includes two different light sources, an array of xenon lamps and two halogen spots, located above and at both sides of the sensory system, respectively. This lighting system is connected to a control unit that enables the independent power on and off of the lamps, and the control of their intensities. Some views of the proposed system are shown in Figure 1.

The RGB camera and multispectral imaging system will provide the input data required for the detection and characterisation of areas of interest that could belong to fruits, whereas the TOF 3D camera will supply simultaneously fast acquisition of accurate distances and intensity images of targets, enabling the localisation of fruits in the coordinate space. Intrinsic and extrinsic calibration parameters of both cameras were estimated by using the Matlab camera calibration toolbox (http://www.vision.caltech.edu/bouguetj/calib_doc/). A distance measurement calibration was also carried out in Matlab (http://www.mathworks.com/products/matlab/) for the TOF camera by following the method proposed in [26].

In order to confer versatility to the set-up, the whole proposed multisensory system is installed on a pan-tilt unit that facilitates the data acquisition of different viewpoints. The tilt movement has a limited angular displacement of α = ±30° relative to the horizontal axis due to mechanical constraints. The yaw movement has no mechanical constraint, so it could rotate 360° around the vertical axis. However, for the stated application, the automatic yaw movement will be restricted for azimuthal angles within the range given by 0° ≤ β ≤ 180°.

**Figure 2.**
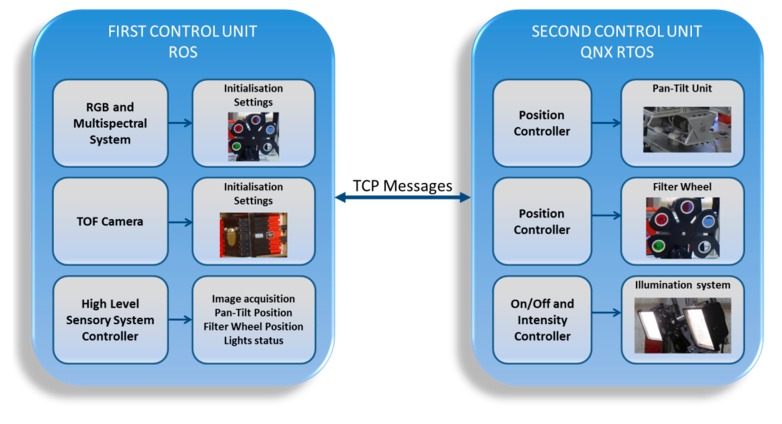
Multisensory system architecture.

The control architecture for the proposed multisensory system consists of two main parts, a unit implemented in Robot Operating System (ROS, http://www.ros.org/), responsible for managing the sensing devices and the high level control of the hardware elements, and a second unit implemented in QNX RTOS (http://www.qnx.com) for the low level control of the hardware elements, which are the motorised filter wheel, the illumination system and the pan-tilt unit (see Figure 2). Thus, the principal functions of the first unit are the initialisation of the CCD and TOF cameras (acquisition mode, pixel format), the setting of the camera parameters according to the working conditions (exposure time in the CCD camera and integration time in the TOF camera) and the control of the image acquisition procedure. Three ROS nodes are developed for achieving these functionalities: one for each camera and the sensory system controller node. Synchronous acquisition of the CDD and TOF camera is achieved when the sensory system controller publishes a trigger message that is sent when the filter wheel reaches a requested position. Immediately after the frame data acquisition is successfully completed, the sensory system controller node sends a command to the second unit implemented in QNX in order to initiate the motion of the filter wheel to the next target position. This node also sends commands for controlling the lights and the pan-tilt unit when required [27].

The second unit is in charge of the low level control for the high accurate positioning of the filter wheel (with a position error of ±0.01285° and a maximum time delay of 50 ms for the positioning of each filter), switch on, switch off and intensity variation of the illumination system, as well as the high accurate positioning of the pan-tilt unit, being the PID controller the preferred option for this purpose. First and second unit communicate between them via TCP messages. These messages contain the parameters and commands required for controlling and monitoring the motion and the data acquisition tasks of the multisensory system.

### 2.2. Pre-Processing Algorithms

Before investigating methodologies and techniques that permit us to detect and locate fruits with high accuracy, it is necessary to count with appropriate pre-processing algorithms that allow us to take full advantage of the data acquired with the designed multisensory system. Taking into consideration the configuration described in the previous subsection, two complementary pre-processing algorithms are proposed: a pixel-based classification algorithm that labels areas of interest that are candidates for belonging to fruits and a registration algorithm that combines the results of the aforementioned classification algorithm with the data provided by the TOF camera for the 3D reconstruction of the desired regions. These algorithms are described below.

Several studies have demonstrated that different targets with a similar appearance when they are captured by an RGB camera can exhibit distinctive properties if they are examined with spectral systems capable of acquiring several separated wavelengths [28]. For this reason, the first algorithm deals with the combination of RGB and filtered images acquired with the proposed multisensory system in order to achieve a classification system capable of distinguishing the different elements of the scene [21]. The algorithm, based on Support Vector Machines (SVMs), is capable of labelling each pixel of the image into four classes that are: stems and branches, fruits, leaves, and background. SVM is a supervised learning method utilized for classifying set of samples into two disjoint classes, which are separated by a hyperplane defined on the basis of the information contained in a training set [29]. In the case at hand, four SVMs are utilized sequentially, each one for detecting a class against the rest. Therefore, after the first SVM is applied, pixels identified as belonging to fruit class are labelled and a mask is generated in such a way that only the remaining pixels are considered for the following SVMs. This step is then repeated for the rest of the classes in the following order: leaves, stems and branches, and finally background. The SVM classifiers are trained by selecting a random subset of samples from the RGB and filtered images and manually labelling the regions of interest from these images into the four semantic classes mentioned above. The algorithm was implemented in C++ with the aid of the Open Source Computer Vision Library (OpenCV) [30,31].

**Figure 3.**
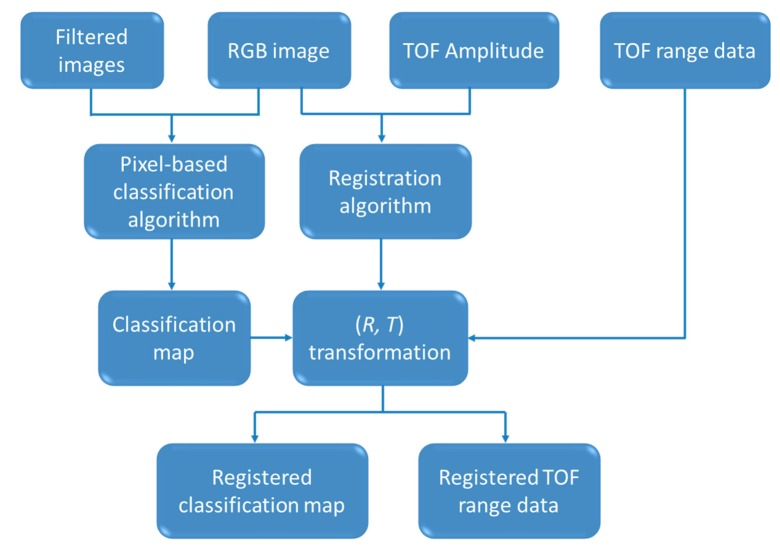
Inputs and outputs of the proposed pre-processing algorithms.

Once regions of interest have been detected in the scene, it is necessary to locate them spatially. The TOF camera included in the proposed multisensory system provides amplitude, depth and confidence data simultaneously for each pixel of the image captured. The amplitude represents the greyscale information, the depth is the distance value calculated within the camera and the confidence is the strength of the reflected signal, which means the quality of the depth measurements. Although TOF data is fundamental for localisation purposes, it is still necessary to automatically match this information with the classification map obtained from the previous step in a common reference frame. For accomplishing this procedure it should be taken into account that TOF images and resulting classification maps come from sensors that exhibit different field of view and different pixel array size. Thus, data will only depict the same content partially, and the pixel correspondence will not be direct. To overcome this problem, the random sample consensus (RANSAC) algorithm is adopted for the multisensory registration. RANSAC is one of the most robust algorithms for model fitting to data containing a significant percentage of errors [32]. This iterative method estimates parameters of a mathematical model from a set of observed data which contains outliers [33]. As the multisensory system has been designed in an enclosure that prevents the relative movements between the different elements that compose it, the idea is to use the RANSAC method to find the rotation and translation (*R, T*) that enable the transformation of the TOF data into the reference frame of the classification map. For that, *N* pairs of control point matches between Frames *F*_1_ and *F*_2_ are selected, where *F*_1_ and *F*_2_ correspond to TOF and RGB frames respectively. Note that the RGB frame is utilised for convenience, as it is consistent with that of the classification map. The control points are represented by 2D coordinates $\left(X_{1}^{i},X_{2}^{i}\right)$ in their respective reference systems. RANSAC samples the solution space of (*R, T*) and estimates its fitness by counting the number of inliers, *f*_0_:

$$f_{0}\left(F_{1},F_{2},R,T\right)=\overset{N}{\underset{i}{\text{∑}}}L\left(X_{1}^{i},X_{2}^{i},R,T\right)$$

where:

$$L\left(X_{1}^{i},X_{2}^{i},R,T\right)=\{\begin{array}{cc} 1, & e=\left\|RX_{1}^{i}+T-X_{2}^{i}\right\|<∊\\ 0, & \text{otherwise} \end{array}$$

and *∊* is the threshold beneath which a features match $\left(X_{1}^{i},X_{2}^{i}\right)$ is determined to be an inlier. RANSAC chooses the transform with the largest number of inlier matches [34]. In this way, the transformation given by (*R, T*) may be applied to any image acquired with the TOF camera, obtaining quickly and efficiently the registered data and it won't be necessary to recalculate this transformation as long as the multisensory rig is not modified. The algorithm for on-line registration of the TOF data with the classification map was implemented in C++. Figure 3 summarises the inputs and outputs of the proposed pre-processing algorithms.

### 2.3. Validation Strategy

The objective of the validation strategy is to establish a structured procedure that provides quantitative information for evaluating the system performance. As it was stated before, harvesting robots require sensory systems that allow reliable detection and localisation of fruits. Thus, the quality of the proposed multisensory system and the associated set of pre-processing algorithms will be rated by comparing the obtained detection and localisation results with ground truth data that will serve as reference. The performance metrics selected for validation purposes are:

- The true positive fruit detection rate, which is a measure of the proportion of the pixels that are correctly identified as belonging to the class fruits. It is defined by:
  $$\text{TP}=\frac{\text{number of pixels of the class fruits correctly classified}}{\text{total number of pixels of the class fruits}}\cdot 100\%$$
- The false positive fruit detection rate, which is the proportion of pixels that are incorrectly classified as belonging to the class fruits. It is calculated as follows:
  $$\text{FP}=\frac{\text{number of pixels incorrectly classified}}{\text{total number of pixels of other classes different to fruits}}\cdot 100\%$$
- The precision of fruit detection, which is a measure of the accuracy. It is defined by:
  $$\text{Precision}=\frac{\text{TP}}{\text{TP}+\text{FP}}\cdot 100\%$$
- The fruit detection error rate, which is given by:
  $$\text{Error rate}=\frac{\text{sum of incorrect classifications}}{\text{total number of classifications}}\cdot 100\%$$
- The mean absolute error [|*e_x_*|, |*e_y_*|, |*e_z_*|] in fruit localisation, which is the average of the absolute differences between the true coordinates of a selected point on the target fruit and the coordinates provided by the TOF camera, both relative to the TOF camera optical centre. The point selected on the fruit for the calculation of the mean absolute error is the centre of the visible outer surface of the fruit.

All these metrics include a clear statement of the end results expected. On the other hand, for the calculation of these performance indicators, two ground truth datasets are required. The first one should contain a list of detectable fruits, as well as their corresponding spatial localisations estimated on the centre of their visible outer surfaces. This first dataset is generated manually by one person. Thus, immediately after the data acquisition and processing of a scene, a human observer, situated in front of the crop, enumerates the visible fruits of the scene and measures their positions in the TOF camera reference frame. In this way, ground truth data generation is conducted under the same practical conditions that the data acquisition and processing. This first ground truth dataset is utilised for estimation of the mean absolute errors in order to evaluate the location capabilities of the proposed system. The second dataset includes a pixel-based masked image for each scene. The masking is performed manually on each RGB image by marking only those pixels that belong to fruits. Thus, second dataset is generated from the images acquired and processed during the experimental tests, and is used for the calculation of the rest of performance indicators with the aim of validating the detection capabilities of the proposed system.

## 3. Experimental Section

In order to evaluate the feasibility of the multisensory system and the associated set of pre-processing algorithms for detecting and locating fruits from different kinds of crops in natural scenarios, an extensive experimental campaign has been conducted in field conditions, in an apple orchard and a vineyard located in Chillan, Chile.

The first phase of the experimental campaign was devoted to the acquisition of training data for the design of the pixel-based classification algorithm. In this case the acquired dataset included RGB and monochrome images with band-pass filters that have centre wavelengths of 635 nm and 880 nm [21]. Since the aforementioned algorithm deals with the classification of each image pixel, each testing set consists of 5,018,400 samples (2448 × 2050 pixels on the image). In order to train the SVMs of the proposed classification algorithm, four acquired datasets were randomly selected. From these RGB and filtered images, representative regions of interest of different sizes were selected for each desired class. Then, the mean reflectance values of these regions were treated as training samples and were manually labelled in four semantic classes: fruits (apples or grapes), stems, leaves and background. With the obtained set of 40 samples per class, the SVMs of the proposed pre-processing algorithm were trained to classify the pixels of the images. The sampling approach for training data could be then considered as a stratified random sampling method, since the population is divided into smaller groups known as strata, which are formed based on members' shared features [35]. Random samples from each stratum are taken, and these subsets are then combined to form the random training sample.

**Figure 4.**
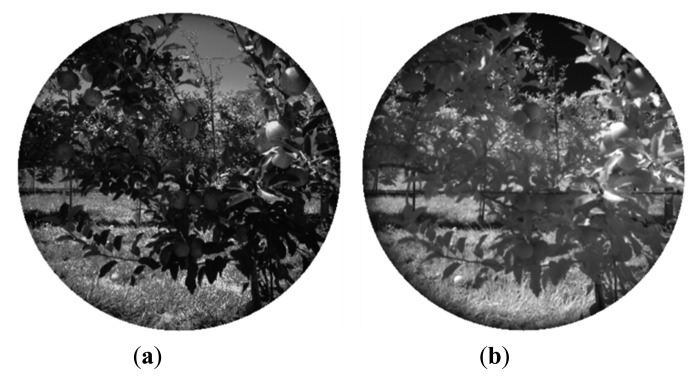
Apple orchard – Filtered images. (**a**) 635 nm image; (**b**) 880 nm image.

**Figure 5.**
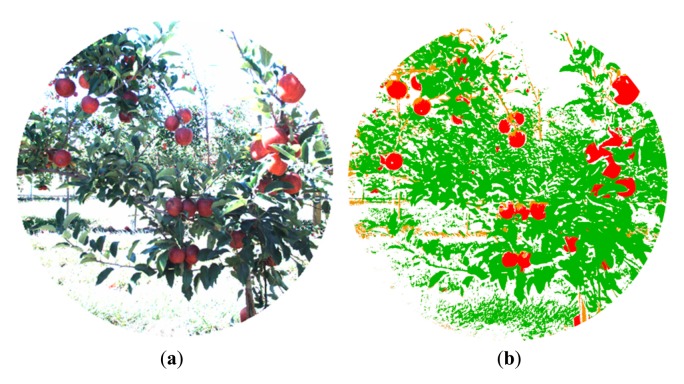
Apple orchard. (**a**) RGB image; (**b**) Classification map.

For the second phase of the experimental campaign, aimed at evaluating the proposed system, the acquired dataset included not only RGB and monochrome filtered images, but also range data. Outputs provided by the proposed system consist of a pixel-based classification map and the TOF (depth and amplitude) registered data. Figures 4 and 5 show the RGB and the filtered images acquired with the multisensory system, as well as the resulting classification map for an apple crop scene.

**Figure 6.**
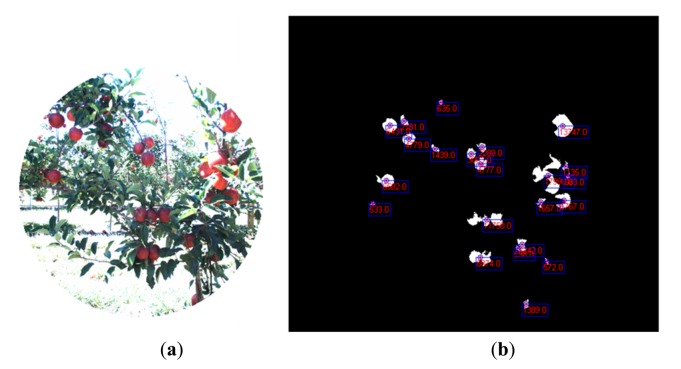
Apple orchard. (**a**) RGB image; (**b**) Centroid and area calculation for each blob that satisfies the predefined requisites to be a fruit candidate for harvesting.

In the classification map, red, brown, green and white colours are utilised to visualize pixels classified as apples, stems and branches, leaves, and background, respectively. Figure 6b illustrates the blobs extracted from the classification map that satisfied the predefined requisites to be a fruit candidate for harvesting in contrast with the original RGB image displayed in Figure 6a. For each blob the centroid and the area are calculated.

**Figure 7.**
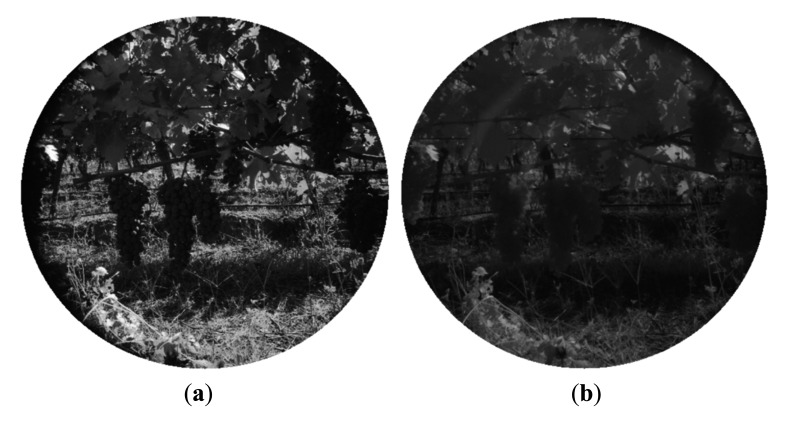
Vineyard – Filtered images. (**a**) 635 nm image; (**b**) 880 nm image.

**Figure 8.**
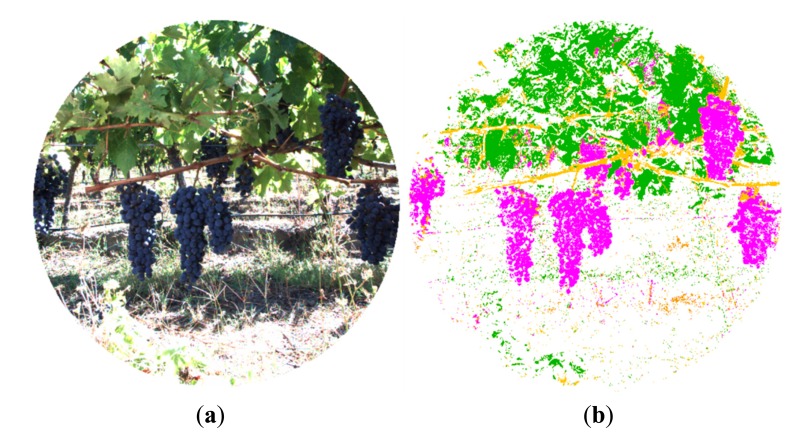
Vineyard. (**a**) RGB image; (**b**) Classification map.

**Figure 9.**
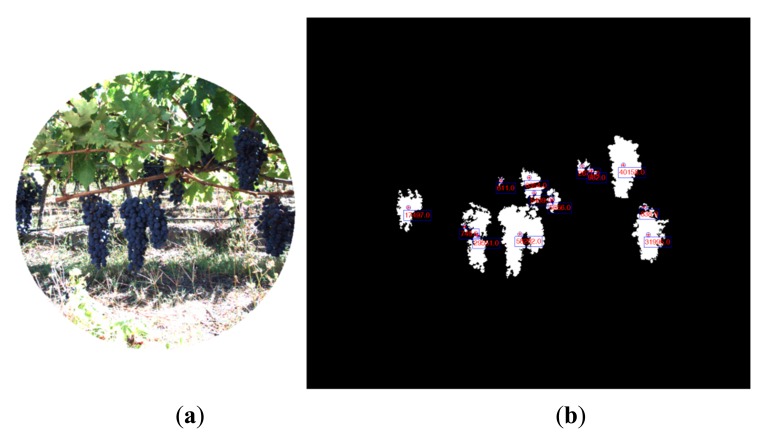
Vineyard. (**a**) RGB image; (**b**) Centroid and area calculation for each blob that satisfies the predefined requisites to be a fruit candidate for harvesting.

In the same manner, Figures 7 and 8 show the RGB and the filtered images acquired with the multisensory system, as well as the resulting classification map for a vineyard scene. In this classification map, magenta, brown, green and white colours are utilised to visualize pixels classified as grapes, stems and branches, leaves, and background, respectively. Figure 9 displays the detected blobs that satisfied the predefined requisites to be a fruit candidate for harvesting.

**Figure 10.**
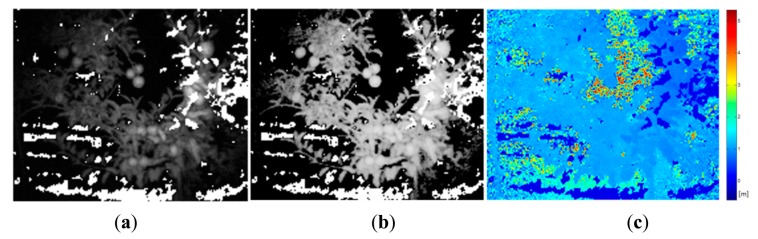
Data acquired with the TOF camera – Apple crop. (**a**) TOF amplitude data; (**b**) TOF camera confidence map; (**c**) Z-axis range data acquired with the TOF camera.

**Figure 11.**
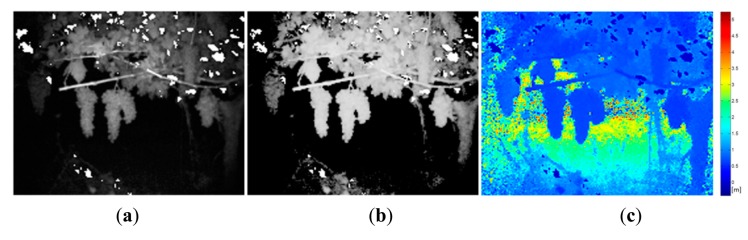
Data acquired with the TOF camera – Vineyard. (**a**) TOF amplitude data; (**b**) TOF camera confidence map; (**c**) Z-axis range data acquired with the TOF camera.

**Figure 12.**
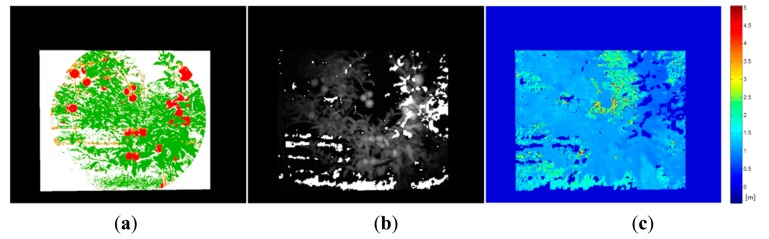
Registered data – Apple crop. (**a**) Registered multispectral map; (**b**) TOF registered amplitude data; (**c**) TOF registered Z-axis range data.

**Figure 13.**
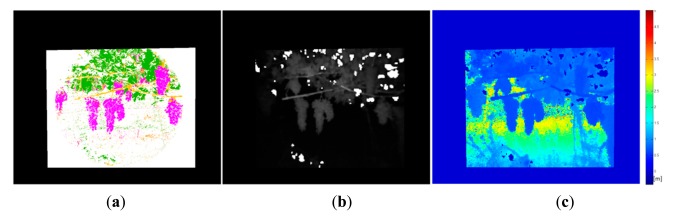
Registered data – Vineyard. (**a**) Registered multispectral map; (**b**) TOF registered amplitude data; (**c**) TOF registered Z-axis range data.

**Figure 14.**
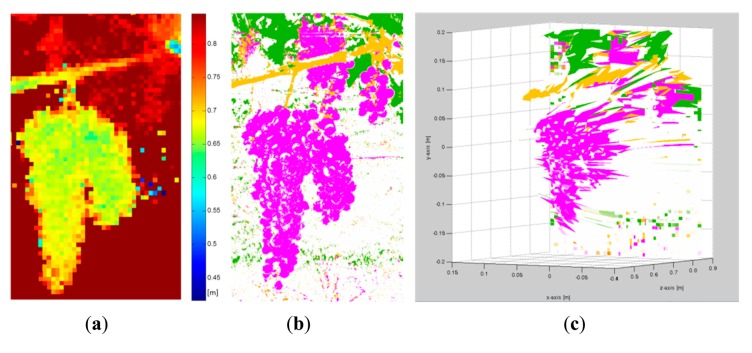
Close-up view of a registered region of interest. (**a**) TOF registered Z-axis range data; (**b**) Registered multispectral map; (**c**) RGB-D visualisation of the registered region of interest.

Once the classification map is obtained, TOF data is registered in order to locate spatially the regions of interest that belong to fruits. Figures 10 and 11 show the original data acquired with the TOF camera for the same scenes presented previously in Figures 4, 5 and 6 and 7, 8 and 9, respectively. The acquired data includes the amplitude (Figures 10a and 11a), the confidence map (Figures 10b and 11b) and the range data in meters (Figures 10c and 11c). Figures 12 and 13 display the resulting multispectral maps, amplitude and range data obtained after applying the registration algorithm. Finally, Figure 14 shows the close-up view of a registered region of interest extracted from the last presented scene.

## 4. Results and Discussion

**Figure 15.**
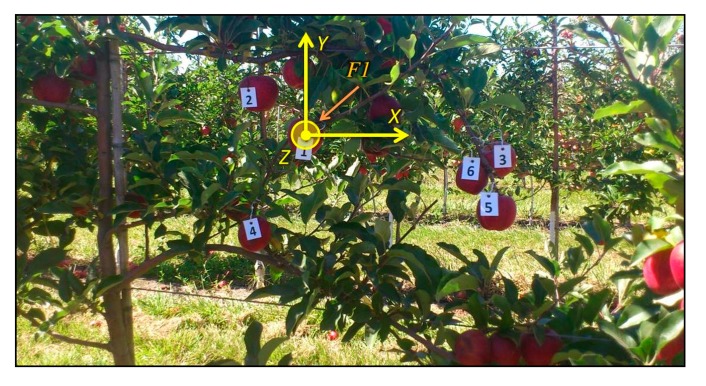
Ground truth data acquisition for a scene of the apple orchard.

**Figure 16.**
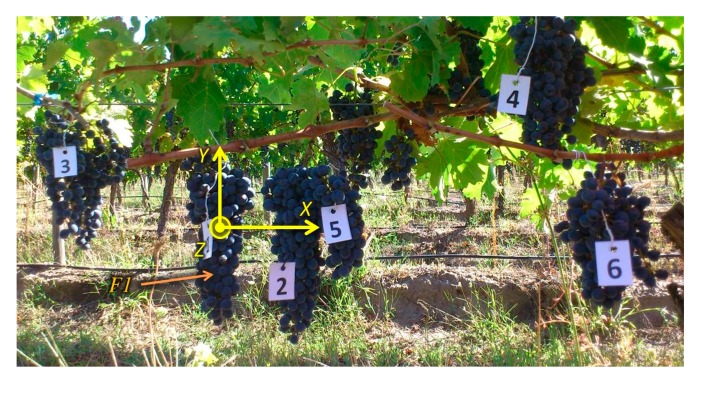
Ground truth data acquisition for a scene of the vineyard.

For validation purposes, a total of 12 scenes from the apple crop and 10 from the vineyard were acquired, processed and evaluated. Ground truth data was carefully collected and produced for each scene in order to carry out a quantitative assessment of the proposed solution. This process involved as first step the manual labelling of some fruits of the scenes acquired and processed during the experimental campaign, as well as the manual measurement of the distance from the frontal plane of the TOF camera to the centre of the visible outer surface of each labelled fruit. Horizontal and vertical distances from a defined reference frame to the centre of the visible outer surface of each labelled fruit were also measured manually. For instance, Figure 15 shows the labelling of one of the scenes acquired in the apple orchard, while Figure 16 displays the same for the vineyard. Note that these images have been acquired with an external camera, different from the RGB camera included in the multisensory rig, only for illustration purposes, and consequently, as can be observed, the point of view is different if they are compared with Figures 5a and 8a. Tables 1 and 2 summarise the ground truth measurements collected for these scenes, where X and Y correspond to the horizontal and vertical distances measured from the origin of the reference frame defined on the image to the centre of the visible outer surface of each labelled fruit, and Z represents the orthogonal distance measured from the frontal plane of the TOF camera to the centre of the visible outer surface of each labelled fruit. The reference frame defined on each image for the ground truth data collection is the centre of the fruit labelled as 1. Thus a transformation of these measurements is required in order to compare them to the data provided by the TOF camera. This transformation only affects to the *x* and *y* coordinates, since *z* coordinate is always referenced to the TOF camera.

After evaluating the data registered from the TOF camera with the collected ground truth, we obtained that the position error ranges from 0 to 4.5 cm in the *x*-axis, from 0 to 6.1 cm in the *y*-axis and from 1 to 7.6 cm in the *z*-axis, with a mean error of 0.8 cm in the *x*-axis, 1.5 cm in the *y*-axis and 2.3 cm in the *z*-axis. Table 3 summarises these results. In natural scenes it is quite easy to find a great number of elements that can affect the response of the TOF camera, which is characterised by suffering from flying pixels, noise and incorrect depth measurements due to the scene geometry and material properties. For instance, the modulated light used by the TOF camera is frequently reflected by multiple surfaces inside the scene before reaching the camera sensor. Border of fruits and leaves produces commonly this kind of multi-path interferences, affecting the range data measurements. Plants elements can also be moved by the wind during the acquisition process, producing erroneous measurements. It has to be considered also that the registration algorithm is dealing with a correspondence between images of 144 × 176 from the TOF camera and images of 2050 × 2480 from the classification maps. Moreover, manual measurement of distances for ground truth data is not exempt from errors, which could explain the appearance of some isolated maximum errors, far from the mean values. Therefore, the mean position errors obtained during the experimental test are quite acceptable bearing in mind the high complexity of the studied scenes and the large difference in the resolution of the TOF images and the classification maps.

**Figure 17.**
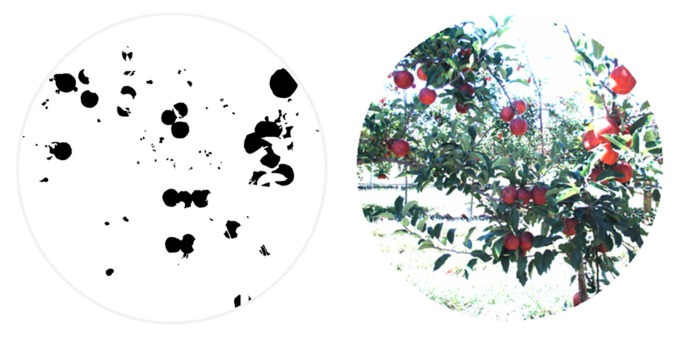
Pixel-based masking for apples.

**Figure 18.**
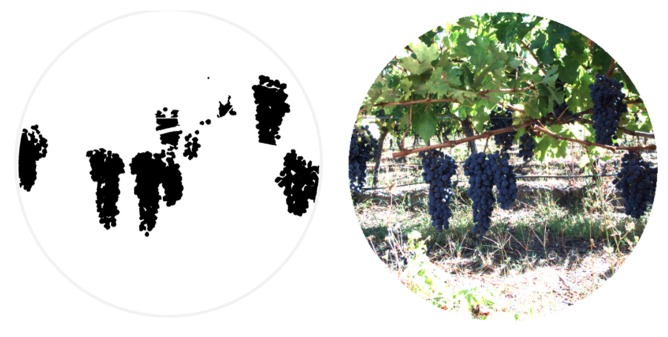
Pixel-based masking for grapes bunches.

The second step implied the pixel-based masking for each fruit of the acquired scenes. This masking was performed manually on five RGB images of the apple orchard and five RGB images of the vineyard by marking only those pixels that belong to the fruits. Figures 17 and 18 show, respectively, the mask images for the apple orchard and vineyard scenes presented before (Figures 5a and 8a). These images are then utilised as ground truth data in the pixel-level comparison carried out with the classification maps obtained from the proposed pre-processing algorithm. Classification performance is then evaluated in terms of true-positive (TP) and false-positive (FP) detections for fruits, precision and total error rate, following the validation strategy described in Subsection 2.3. Results of this evaluation are gathered in Tables 4 and 5. It is important to mention that these results were obtained without carrying out any morphological operation on the classification maps.

Classification of grapes exhibits a slightly lower performance than classification of apples. This may be due to the fact that bunches of grapes present a complex characteristic shape that makes them more prone to shadows and specular reflexions, and consequently more prone to suffering from misclassifications. Ground truth labelling is also more complex for grapes than for apples, which may also contribute to degrade overall performance results.

Nevertheless, high true positive rates are achieved for both grapes and apples detection, reinforced by the low false positive rates. Mean classification precisions were of 99.8% for apples and 97.6% for grapes, whereas mean error rates were of 0.18% and 1.78% for apples and grapes, respectively. Therefore, the proposed pre-processing algorithm attains a high level of correctness in classifying the pixels of images that belong to the target fruits.

## 5. Conclusions

This paper proposes a modular and easily adaptable multisensory system and a set of associated pre-processing algorithms for the detection and localisation of fruits from different kinds of crops. The solution includes a colour camera and a multispectral system for acquiring reflectance measurements in the visible and NIR regions that are used for finding areas of interest that belong to the fruits, and a TOF camera that provides fast acquisition of distances enabling the localisation of the targets in the coordinate space.

The pre-processing algorithms designed for the proposed multisensory system include a classification algorithm based on SVMs that identifies pixels that belong to fruits and a registration algorithm that combines the results of the aforementioned classification algorithm with the data provided by the TOF camera in order to obtain a direct correspondence among their pixels, so range data can be associated to pixels labelled as fruit. An extensive experimental campaign was carried out in order to assess the proposed solution, including the acquisition of not only test data but also training and ground truth data.

In spite of the challenging scenarios found in natural environments, the proposed solution exhibited a satisfactory performance. Multisensory system provides all the data required for detecting and locating fruits, showing a great versatility in dealing with different crops. The pre-processing algorithm based on SVM classifiers affords an accurate enough discrimination of apple tree and vineyard elements, without any pre-treatment of the images, and without any preparation of the crops. Finally, registration algorithm allows the spatial localisation of the regions of interest classified as fruits with enough accuracy.

## Figures and Tables

**Table 1.** Ground truth measurements for the scene presented in Figure 15.

**REFERENCE FRAME – CENTRE OF THE FRUIT 1**			

**FRUIT**	**X [mm]**	**Y [mm]**	**Z [mm]**
1	0	0	1040
2	−125	80	1077
3	345	−40	1053
4	−140	−175	1071
5	340	−130	983
6	290	−70	1026

**Table 2.** Ground truth measurements for the scene presented in Figure 16.

**REFERENCE FRAME – CENTRE OF THE FRUIT 1**			

**FRUIT**	**X [mm]**	**Y [mm]**	**Z [mm]**
1	0	0	598
2	9	−3	585
3	−25	4	682
4	36	12	589
5	15	−2	603
6	42	−4	571

**Table 3.** Position errors from the TOF registered data.

**Axis**	**Minimum Error [cm]**	**Maximum Error [cm]**	**Mean Absolute Error [cm]**
X	0	4.5	0.8
Y	0	6.1	1.5
Z	1	7.6	2.3

**Table 4.** Performance evaluation for apples orchard scenes.

**Scene**	**TP [%]**	**FP [%]**	**Precision [%]**	**Error Rate [%]**
1	97.0	0.17	99.8	0.16
2	91.9	0.39	99.6	0.37
3	98.1	0.09	99.9	0.08
4	97.8	0.20	99.8	0.19
5	98.3	0.11	99.9	0.10
Mean values	96.6	0.19	99.8	0.18

**Table 5.** Performance evaluation for vineyard scenes.

**Scene**	**TP [%]**	**FP [%]**	**Precision [%]**	**Error Rate [%]**
1	89.9	1.28	98.6	1.13
2	82.1	1.59	98.1	1.46
3	79.6	1.65	98.0	1.52
4	80.6	2.82	96.6	2.47
5	83.2	2.70	96.9	2.33
Mean values	83.1	2.01	97.6	1.78
